# Antibiotic Resistance, Virulence Genes, and Molecular Diversity of Clinical *Klebsiella pneumoniae* Isolates from Patients of District Hospital in Central Poland

**DOI:** 10.3390/pathogens14070648

**Published:** 2025-06-30

**Authors:** Barbara Kot, Małgorzata Witeska, Piotr Szweda, Małgorzata Piechota, Elżbieta Kondera, Elżbieta Horoszewicz, Izabela Balak, Ahmer Bin Hafeez, Alicja Synowiec

**Affiliations:** 1 Institute of Biological Sciences, Faculty of Exact and Natural Sciences, University of Siedlce, 14 Bolesława Prusa Str., 08-110 Siedlce, Poland; malgorzata.piechota@uws.edu.pl (M.P.); elzbieta.kondera@uws.edu.pl (E.K.); izabalak@wp.pl (I.B.); 2 Department of Animal Environment Biology, Institute of Animal Science, Warsaw University of Life Sciences—SGGW, Ciszewskiego 8, 02-786 Warsaw, Poland; malgorzata_witeska@sggw.edu.pl; 3 Department of Pharmaceutical Technology and Biochemistry, Faculty of Chemistry, Gdańsk University of Technology, 11/12 G. Narutowicza Str., 80-233 Gdańsk, Poland; piotr.szweda@pg.edu.pl (P.S.); ahmer.bin.hafeez@pg.edu.pl (A.B.H.); 4 Institute of Animal Science and Fisheries, University of Siedlce, 12 Bolesława Prusa Str., 08-110 Siedlce, Poland; elzbieta.horoszewicz@uws.edu.pl; 5 Department of Food Biotechnology and Microbiology, Institute of Food Sciences, Warsaw University of Life Sciences—SGGW, Nowoursynowska 159c, 02-776 Warsaw, Poland; alicja_synowiec@sggw.edu.pl

**Keywords:** β-lactamase, efflux pump, aerobactin, urease, ERIC-PCR, REP-PCR

## Abstract

In hospital environments, pathogenic bacteria spread easily and acquire virulence and antibiotic resistance genes. The aim of the study was an evaluation of the genetic diversity of 109 *K. pneumoniae* isolates recovered from patients of a district hospital in central Poland. The frequencies of genes coding for β-lactamases, efflux pumps, and virulence factors were determined. Genotyping of the isolates was performed with ERIC (Enterobacterial Repetitive Intergenic Consensus) and REP (Repetitive Element Sequence Based) PCR techniques, with 21 and 19 genotypes being identified, respectively. The *bla*_SHV-1_ (92.7%)*, bla*_CTX-M_ group 1 (83.5%), *bla*_TEM-1_ (28.4%), *bla*_NDM-1_ (16.5%)*, bla*_VEB-1_ (11.0%), *bla*_CTX-M_ group 9 (3.7%), *bla*_KPC_ (1.8%), *bla*_IMP_, *bla*_OXA-48_, *bla*_CTX-M_ group 2, *bla*_CTX-M_ groups 8, and 25/26 (0% each) and efflux pumps: *AcrAB* (100%)*, tolC* (93.6%)*,* and *mdtk* (60.5%), and virulence genes coding: urease subunit *ureA* (94.5%) endotoxins *wabG* (92.7%) and *uge* (64.2%), and siderophore *iucB* (3.7%) were detected. The *bla*_SHV-1_, *bla*_CTX-M_ group 1, *mdtk*, *tolC*, *AcrAB* (16.5%); *bla*_SHV-1_, *bla*_CTX-M_ group 1, *tolC*, *AcrAB* (15.6%), and *bla*_SHV-1_, *bla*_CTX-M_ group 1, *bla*_NDM-1_, *mdtk*, *tolC*, *AcrAB* (11.9%) were the most common resistance patterns. The distribution of resistance and virulence genes varied more between hospital wards than between different clinical materials. Hospital’s antibiotic-resistant and virulent *K. pneumoniae*, able to spread among humans, animals, and in the environment, pose a significant threat to public health.

## 1. Introduction

*Klebsiella pneumoniae* is an opportunistic pathogen that spreads worldwide, causing nosocomial and community-acquired infections [1]. These bacteria can colonize mucosal surfaces without causing infections and may disseminate from mucosae to other parts of the host organism, causing serious diseases. Among these, the most frequent are pneumonia, sepsis, bloodstream infections, pyogenic liver abscesses, meningitis, wound infections, and urinary tract infections (UTIs) [2,3]. The susceptibility to *K. pneumoniae* infection depends on the virulence and antibiotic resistance of the isolates, as well as host factors such as genetics, age, and immune status [4]. Besides the classical *K. pneumoniae* strains that cause serious infections (meningitis, pneumonia, UTIs, bacteremia) in the immunocompromised host, hypervirulent *K. pneumoniae* (hvKP) strains have emerged, capable of infecting both healthy and immunocompromised individuals. These infections tend to be highly invasive because hvKP can establish infections in the liver and cause pneumonia, lung abscesses, and other complications [5]. In hospitalized patients, *K. pneumoniae* infections often result from contamination in the hospital environment, as the bacteria can colonize medical equipment and devices [6] and may be transmitted through the hands of medical staff and their contact with patients [7]. The mortality rate of hospital-acquired pneumonia caused by *K. pneumoniae* remains over 50% [8]. Due to the increasing antibiotic resistance, *K. pneumoniae* is one of the most dangerous pathogens causing life-threatening infections. β-lactams, including penicillins, cephalosporins, and carbapenems, are widely used due to their therapeutic efficacy and wide range of options [9]. However, *K. pneumoniae* exhibits various antibiotic resistance mechanisms, and resistance to β-lactams, particularly carbapenems, makes treatment especially challenging because of the production of extended-spectrum β-lactamases (ESBLs) and carbapenemases [7]. According to the WHO (World Health Organization), ESBL-producing and carbapenem-resistant *K. pneumoniae* pose significant public health threats [10]. Ambler et al. [11] classified β-lactamases into four classes, A to D, based on their amino acid sequences. A, C, and D classes include enzymes with an active-site serine, while class B includes a heterogeneous group of zinc metalloenzymes (metallo-β-lactamases—MBLs). Class A includes TEM (active against aminopenicillins and cephalosporins), SHV (sulfhydryl variant) with similar activity to TEM, CTX-M (cefotaximase enzyme), and KPC (*K. pneumoniae* carbapenemase). Point mutations that enabled TEM and SHV to hydrolyze oxyiminocephalosporins, such as cefotaxime and ceftazidime, resulted in the emergence of the “extended-spectrum” phenotype (extended-spectrum beta-lactamases—ESBLs). VIM (Verona imipenemase), IMP (Imipenemase), and NDM (New Delhi metallo-β-lactamases), belonging to class B, hydrolyze the β-lactam antibiotics, including last-generation cephalosporins and carbapenems. AmpC enzymes (class C) cause resistance to most penicillins, cephalosporins, cephalomycin, and β-lactamase inhibitor-β-lactam combinations. OXA enzymes belonging to class D showed activities against the semisynthetic penicillin oxacillin, cephalosporins, and carbapenems such as ceftazidime and cefotaxime. To better understand this pathogen, in our previous study, we evaluated the presence of virulence genes encoding fimbriae, siderophores, capsules, and hypercapsules, and also susceptibility to selected antibiotics of *K. pneumoniae* isolates causing various infections in hospitalized patients in a district hospital in central Poland. We showed that many nosocomial *K. pneumoniae* isolates were multidrug-resistant (MDR) (68.8%) and produced ESBLs (59.6%). In some isolates, genes associated with hypervirulence of *K. pneumoniae* were also revealed [12].

It is very important to know the potential virulence of *K. pneumoniae*, which significantly affects the course and severity of the infection. On the other hand, identifying antibiotic resistance mechanisms in a situation of the rapid spread of resistance genes among *K. pneumoniae* isolates becomes crucial for effective treatment of infections that often threaten patients’ lives. Due to the growing threat from this microorganism, we undertook further studies using the same *K. pneumoniae* isolates analyzed in our previous work [12].

The aim of the current study was to assess the occurrence of genes encoding resistance to β-lactam antibiotics and efflux pumps. To expand knowledge of the virulence factors of *K. pneumoniae* that are important in the development of infection, we also evaluated the occurrence of additional genes associated with the virulence of these isolates that were not assessed in our previous study [12].

We also determined the degree of relatedness of isolates from various hospital wards and clinical materials. The quantitative analysis included the genes associated with virulence and hypervirulence of *K. pneumoniae* identified in the previous study to characterize all isolates completely.

## 2. Materials and Methods

### 2.1. Bacterial Isolates

The study included 109 *K. pneumoniae* isolates from patients hospitalized in a district hospital in central Poland (Our Lady of Perpetual Help Hospital, Wołomin). The isolates were collected from 2018 to 2022 as part of routine diagnostic microbiology conducted in this hospital. Based on the Medical Microbiological Laboratory database, only one *K. pneumoniae* isolate per patient was included in the study. The methods of isolation and identification have been described previously [12]. The isolates were collected from the patients in 11 hospital wards: Internal (31 isolates, 28.4%), Nephrology (20 and 18.4%), Intensive Care (15 and 13.8%), Neurology (14 and 12.8%), Surgery (13 and 11.9%), Orthopedics (3 and 2.8%), Urology (2 and 1.8%), Dialysis Station, Emergency Department, Gynecology and Obstetrics, and Pediatrics (1 and 0.92% each). For seven isolates (6.4%), no information about the ward of origin was available. Most isolates were obtained from urine (39; 35.8%), blood (22; 20.2%), anus (17; 15.6%), respiratory tract: bronchial aspirate (10), bronchoalveolar lavage (3), and endotracheal tube (3), totaling 16 isolates (14.7%). Wound samples accounted for 13 isolates (11.9%), and only two isolates (1.8%) were collected from other materials.

### 2.2. DNA Isolation

Genomic DNA from bacterial cells was isolated using the NucleoSpin Microbial DNA kit (Macherey-Nagel GmbH & Co. KG, Düren, Germany) according to the manufacturer’s protocol.

### 2.3. Detection of Virulence, β-Lactamases, and Efflux Pump Genes

Primer sequences specific for the identified virulence genes (*iucB*, *wabG*, *uge*, *urea);* genes conferring resistance to β-lactams (*bla*_NDM-1_, *bla*_IMP_, *bla*_KPC_, *bla*_OXA-48_, *bla*_CTX-M_ group 1, *bla*_CTX-M_ group 2, *bla*_CTX-M_ groups 8 and 25/26, *bla*_CTX-M_ group 9, *bla*_SHV-1_, *bla*_VEB-1_, *bla*_TEM-1_); and efflux pumps gene (*AcrAB*, *tolC*, and *mdtk*), synthesized at the Institute of Biochemistry and Biophysics, Polish Academy of Sciences (Warsaw, Poland), are listed in Supplementary Table S1.

Simplex PCR for each gene, except *bla*_CTX-M_ genes, was performed in a 25 µL volume containing 1 µL of DNA template, 12.5 µL of the PCR Mix Plus HGC (0.1 U/µL of *Taq* DNA polymerase, 4 mM of MgCl_2_, 0.5 mM of each dATP, dCTP, dGTP and dTTP) (A&A Biotechnology, Gdynia, Poland) and gene-specific primers at a final concentration of 200 nM.

Thermal cycling conditions were as follows: pre-denaturation at 95 °C for 4 min; 35 cycles of denaturation at 95 °C for 0.5 min, primer annealing at 58 °C for 0.5 min, and extension at 72 °C for 1 min. Amplification was carried out in the Eppendorf Mastercycler Nexus Gradient (Hamburg, Germany). Negative controls included all PCR components except template DNA. Positive controls included the genomic DNA from the isolates in which the target genes had previously been identified. These controls were included in each test run.

*bla*_CTX-M_ genes were detected by multiplex PCR according to Xu et al. [13]. The PCR products were analyzed on electrophoresis in 1.5% agarose gels stained with ethidium bromide. Molecular size markers (Merck Life Science, Poznan, Poland) were also run alongside samples to verify product sizes. Gels were electrophoresed in a 2×Tris-borate buffer at 70 V for 1.5 h. The PCR amplicons were visualized using UV light (Syngen Imagine, Syngen Biotech, Wrocław, Poland).

### 2.4. ERIC-PCR (Enterobacterial Repetitive Intergenic Consensus Polymerase Chain Reaction) Typing

PCR was performed in a 25 µL volume containing 1 µL of DNA template, 12.5 µL of the PCR Mix Plus HGC (0.1 U/µL of *Tag* DNA polymerase, 4 mM of MgCl_2_, 0.5 mM of each dATP, dCTP, dGTP, and dTTP) (A&A Biotechnology, Gdynia, Poland), and specific primers at a final concentration of 200 nM (ERIC1: 5′-CACTTAGGGGTCCTCGAATGTA-3′; ERIC2: 5′-AAGTAAGTGACTGGGGTGAGCG-3′) [14]. Thermal cycling conditions were pre-denaturation at 94 °C for 3 min, 35 cycles of denaturation at 94 °C for 1 min, primer annealing at 52 °C for 1 min, extension at 72 °C for 2 min, and a final extension for 5 min at 72 °C.

### 2.5. REP-PCR Typing

DNA was subjected to PCR using the primers REP 1R-I (5′-IIIICGICGICATCIGGC-3′) and REP 2-I (5′-ICGICTTATCIGGCCTAC-3′) as described by Versalovic et al. [13]. Amplification reactions were performed in a final volume of 25 µL containing 1 µL of DNA template, 12.5 µL of the PCR Mix Plus HGC (0.1 U/µL of *Tag* DNA polymerase, 4 mM of MgCl_2_, 0.5 mM of each dATP, dCTP, dGTP, and dTTP) (A&A Biotechnology, Gdynia, Poland), and primers at concentrations of 200 nM.

The thermal cycling conditions were pre-denaturation at 95 °C for 6 min, 30 cycles of denaturation at 94 °C for 1 min, primer annealing at 40 °C for 1 min, extension at 65 °C for 8 min, and a final extension at 65 °C for 16 min [15].

### 2.6. Electrophoresis and Computer Analysis

The amplification products were electrophoresed in a 1.5% agarose gel in 2×Tris-borate buffer at 70 V for 2 h. GeneRuler™ 100 bp Plus DNA Ladder (Thermo Fischer Scientific, Waltham, MA, USA) was used as the DNA standard. Bands were visualized under UV light (Syngen Imagine, Syngen Biotech, Wrocław, Poland). The images were processed, and band sizes (+/− 5 bps) were estimated with Image Lab Software version 5.2.1 (Bio-Rad Laboratories Inc., Hercules, CA, USA). The resulting fingerprints were analyzed by MVSP software version 3.21, and dendrograms were generated using nearest-neighbor cluster analysis.

### 2.7. Statistical Analysis

Data were analyzed using Statistica 13 (TIBCO Software Inc., Camino Ramon, CA, USA) and R (Free Software Foundation’s GNU General Public License). Analysis of the differences in frequency of the presence of each gene between the wards (only those that included ≥5 cases: Internal, Nephrology, Intensive Care, Neurology, and Surgery) and materials was performed using Pearson’s chi-squared test. The same test was used to evaluate differences in the frequencies of ERIC and REP types between the wards and materials; only types represented by ≥5 isolates (E1–E4 and R1–R6) were included in the analysis. Spearman’s correlation analysis was used to examine relationships between the occurrences of all studied genes.

For analysis of the quantitative data (total number of resistance and virulence genes per isolate), Pearson’s correlation coefficients were calculated to explore relationships between resistance and virulence. ANOVA with post hoc Tukey’s Honest Significant Difference (HSD) test for unequal sample sizes was used to assess the significance of differences in the numbers of resistance and virulence genes among isolates from different wards and materials. *p* ≤ 0.05 was assumed to be a significant level for all analyses.

The heatmap was generated using R version 4.1.1. Hierarchical clustering was performed using the Unweighted Pair Group Method with Arithmetic Mean (UPGMA) as the linkage method and Euclidean distance as the dissimilarity measure.

For quantitative analysis (average numbers of resistance and virulence genes in each isolate) and the heatmap, virulence genes (*fimH*, *mrkD*, *clbA*, *clbB*, *entB*, *irp-1*, *kfu*, *wcaG*) and genes associated with hypervirulence of *K. pneumoniae* (*rmpA*, *iroD*, *iroN*) identified in the previous study [12] were included to provide a complete characterization of all isolates.

## 3. Results

### 3.1. The Presence of Genes Encoding β-Lactam Resistance, Efflux Pumps, and Virulence in K. pneumoniae Isolates

In the studied *K. pneumoniae* isolates, the following β-lactamase genes were not detected: *bla*_OXA-48_, *bla*_IMP_, and *bla*_CTX-M_ groups 2, 8, and 25/26. These genes were therefore excluded from the analysis, tables, and heatmap. Among the β-lactamases genes detected in all 109 *K. pneumoniae* isolates, *bla*_SHV-1_ was the most frequently detected, being present in 92.7% of isolates. The *bla*_CTX-M_ group 1 gene was also common (83.5%), whereas *bla*_CTX-M_ group 9 was detected only in 3.7% of isolates. The *bla*_TEM-1_ gene was detected in 28.4% of isolates, while *bla*_NDM-1_ and *bla*_VEB-1_ genes were present in 16.5% and 11.0% of isolates, respectively. The *bla*_KPC_ gene was detected only in two isolates (1.8%).

The distribution of β-lactamase genes among *K. pneumoniae* isolates from various clinical materials and hospital wards is presented in Table 1.

Comparison of these data revealed statistically significant differences in the occurrence of these genes between the wards and materials. The *bla*_NDM-1_ gene was significantly more frequent in the isolates from the Surgery (*p* = 0.008), Intensive Care (*p* = 0.002), and Neurology (*p* = 0.030) wards compared to Nephrology, and more frequent in Intensive Care isolates than in those from the Internal ward (*p* = 0.015). The *bla*_TEM-1_ gene was significantly more frequent in Nephrology isolates compared to Neurology (*p* = 0.013) and Internal ward (*p* = 0.008). Additionally, *bla*_VEB-1_ occurred significantly more often in *K. pneumoniae* obtained from Nephrology compared to Intensive Care (*p* = 0.048) and Internal ward (*p* = 0.002).

The frequencies of various genes also differed between the groups of isolates from different materials. The analysis revealed that the *bla*_NDM-1_ gene was more frequent in blood isolates than in urine (*p* = 0.033), and in isolates from the anus compared to those from urine (*p* = 0.000), blood (*p* = 0.009), and wounds (*p* = 0.004). The gene *bla*_TEM-1_ was more frequently found in urine isolates compared to blood (*p* = 0.010) and anus (*p* = 0.014), while *bla*_VEB-1_ was more common in urine isolates than in those from the respiratory tract (*p* = 0.036) and anus (0.031). The frequency of the isolates with other β-lactamase genes in clinical materials and hospital wards did not differ significantly.

Among the genes coding efflux pumps in *K. pneumoniae* isolates, the AcrAB-TolC pumps were identified in 93.6% of isolates; however, in seven isolates with the *AcrAB* gene, the *tolC* gene coding an outer-membrane channel (TolC) was not detected. The prevalence of the *mdtk* (multidrug efflux pump system) gene (60.5%) was lower than the *AcrAB* (Table 2).

The *mdtk* gene was more frequent in urine isolates compared to those from blood (*p* = 0.013) and wounds (*p* = 0.010), and in respiratory tract isolates compared to wound isolates (*p* = 0.047).

**Figure 1:**
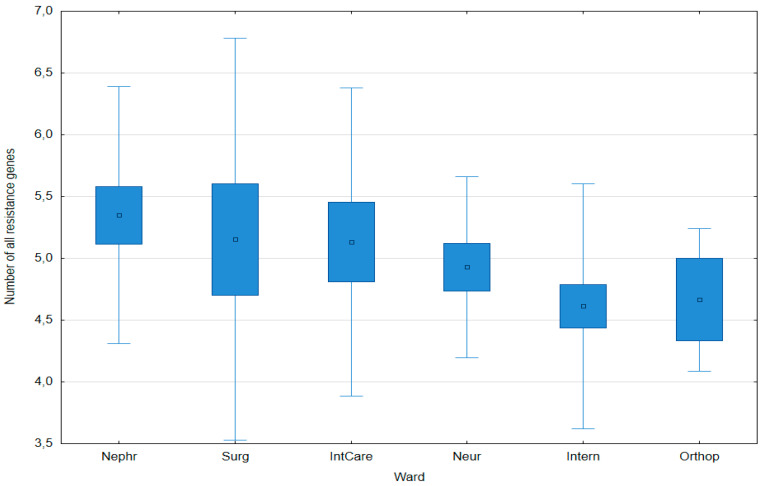
Number of all resistance genes (sum of genes encoding β-lactamases and efflux pumps) in *Klebsiella pneumoniae* isolates obtained from various hospital wards. Data are presented as arithmetic means (point), standard error: S.E. (box), and standard deviation: S.D. (whiskers); no significant differences were found (post hoc Tukey’s Honest Significant Difference (HSD) test, *p* ≤ 0.05).

The average number of all resistance genes (sum of genes encoding β-lactamases and efflux pumps) did not differ significantly between hospital wards (Figure 1).

**Figure 2:**
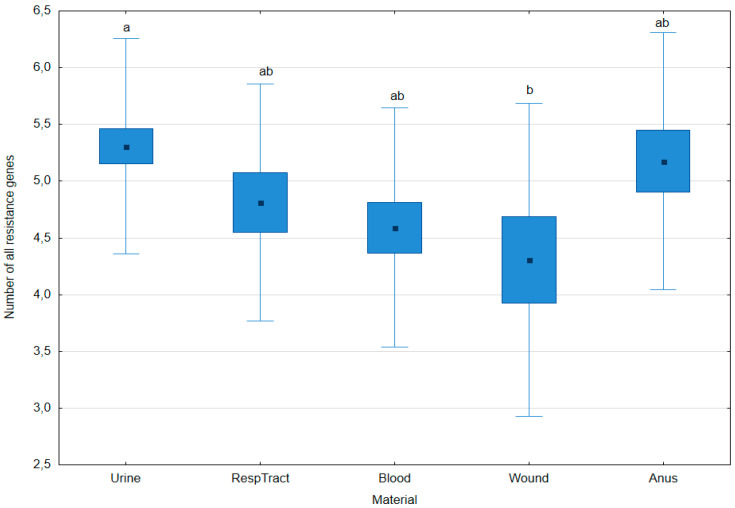
Number of all resistance genes (sum of genes encoding β-lactamases and efflux pumps) in *Klebsiella pneumoniae* isolates obtained from various clinical materials. Data are presented as arithmetic means (point), standard error: S.E. (box), and standard deviation: S.D. (whiskers); significant differences are indicated by different letter superscripts (post hoc Tukey’s Honest Significant Difference (HSD) test, *p* ≤ 0.05).

The average number of all resistance genes in urine isolates was higher (*p* = 0.048) than in wound isolates. The presence of all resistance genes in isolates from other clinical materials did not differ significantly (Figure 2).

The average number of resistance genes encoding β-lactamases and efflux pumps did not differ significantly between hospital wards (Figure S1) and clinical materials (Figure S2).

The prevalence and distribution of virulence genes are shown in Table 3.

Endotoxin-related genes: *wabG* and *uge* were detected in 92.7% and 64.2% of isolates, respectively. The *ureA* gene encoding the urease subunit was present in 94.5% of isolates.

The iron siderophore aerobactin synthase gene (*iucB*) was detected in only 3.7% of isolates from blood and wounds. The gene *iucB* was more frequently observed in wound isolates than in blood (*p* = 0.012).

To provide a comprehensive characterization of all isolates, the genes associated with virulence (*fimH*, *mrkD*, *clbA*, *clbB*, *entB*, *irp-1*, *kfu*, *wcaG*) and hypervirulence of *K. pneumoniae* (*rmpA*, *iroD*, *iroN*) identified in a previous study [12] were included in the quantitative analysis.

**Figure 3:**
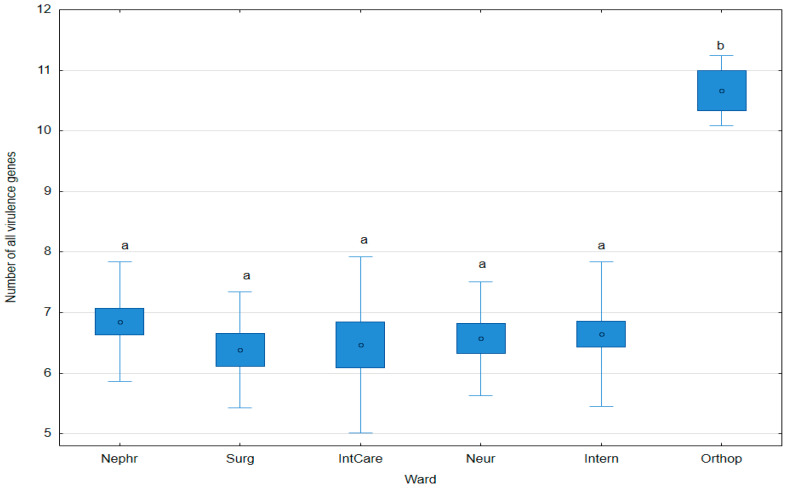
Number of all virulence genes (sum of genes encoding non-hypervirulence and hypervirulence mechanisms) in *Klebsiella pneumoniae* isolates obtained from various hospital wards. Data are presented as arithmetic means (point), standard error: S.E. (box), and standard deviation: S.D. (whiskers); significant differences are indicated by different letter superscripts (post hoc Tukey’s Honest Significant Difference (HSD) test, *p* ≤ 0.05).

The average number of all virulence genes (sum of genes encoding both non-hypervirulence and hypervirulence mechanisms) was significantly higher in Orthopedics isolates compared to the other wards (*p* = 0.001–0.003) (Figure 3).

**Figure 4:**
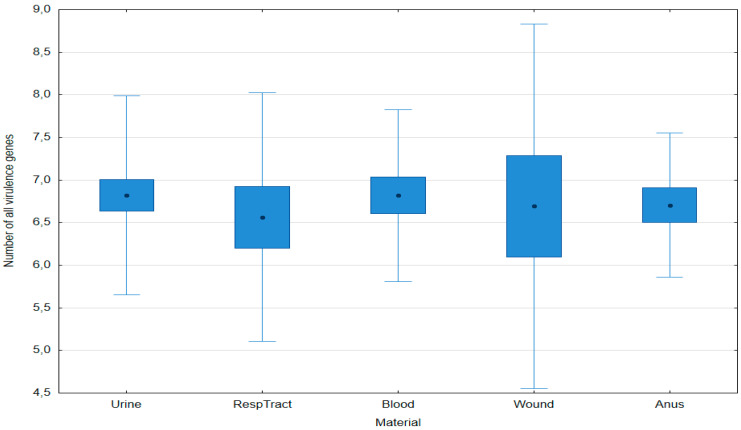
Number of virulence genes (sum of genes encoding non-hypervirulence and hypervirulence mechanisms) in *Klebsiella pneumoniae* isolates obtained from various clinical materials. Data are presented as arithmetic means (point), standard error: S.E. (box), and standard deviation: S.D. (whiskers); no significant differences found (post hoc Tukey’s Honest Significant Difference (HSD) test, *p* ≤ 0.05).

The average number of all virulence genes did not significantly differ between the isolates from various materials (Figure 4).

The number of hypervirulence genes in *K. pneumoniae* isolates was significantly higher in Orthopedics isolates (*p* = 0.000) compared to the other wards (although only three isolates from this ward were analyzed) (Figure S3). In contrast, the number of genes encoding non-hypervirulence and hypervirulence mechanisms did not differ significantly between the isolates from various materials (Figure S4).

The correlation analysis (Pearson’s) revealed a significant but weak relationship (r = 0.329) between the total number of resistance and non-hypervirulence genes. Weak correlations were observed between the number of genes coding efflux pumps and all virulence genes (r = 0.252), and efflux pump genes and non-hypervirulence genes (r = 0.327). The correlation analysis (Spearman’s) showed significant strong relationships between the occurrence of the following gene pairs: *tolC* and *wabG* (r = 0.931), *tolC* and *ureA* (r = 0.921), *wabG* and *ureA* (r = 0.858), and *iucB* and *rmpA* (0.745).

### 3.2. ERIC- and REP-PCR Typing

**Figure 5:**
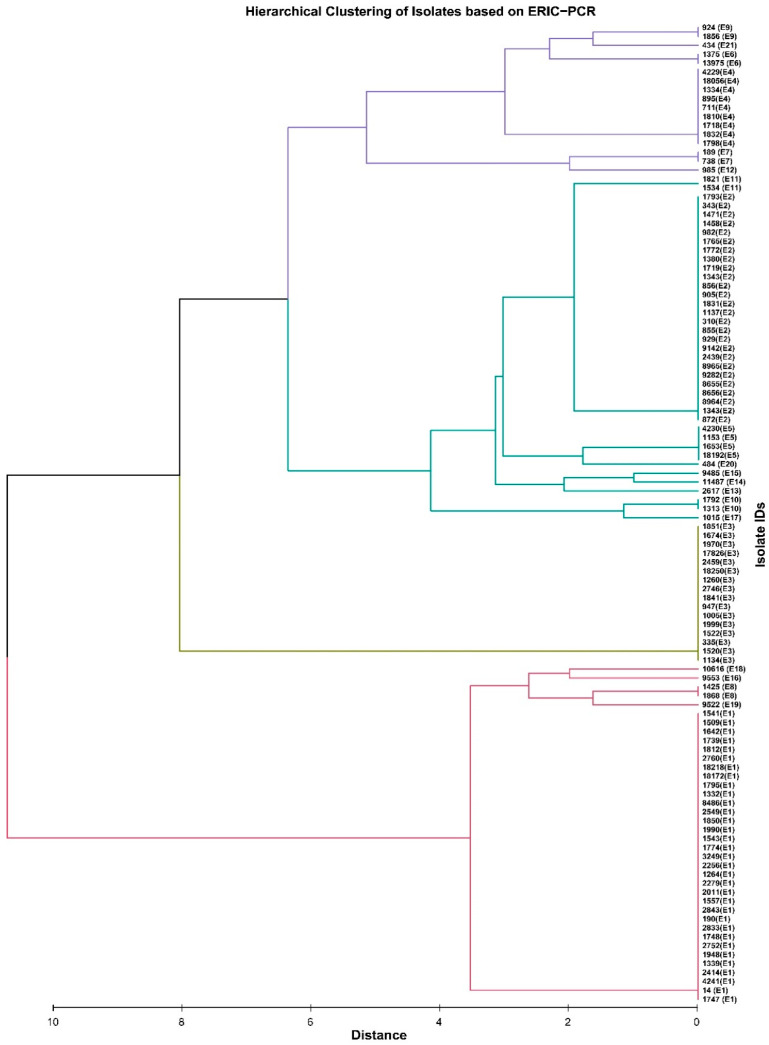
Dendrogram showing the relationship of *K. pneumoniae* isolates based on ERIC-PCR.

All 109 *K. pneumoniae* isolates were characterized by ERIC-PCR and REP-PCR to determine their molecular fingerprinting and phylogenetic relationships. ERIC-PCR generated banding patterns ranging from 0.3 to 2.5 kb. *K. pneumoniae* isolates showed considerable diversity because the ERIC-PCR categorized these isolates into 21 unique ERIC profiles (E types, designated as E1 to E21). Of the 109 isolates, 33 (30.3%) belonged to E1, 25 (22.9%) to E2, 16 (14.7%) to E3, 9 (8.3%) to E4, 4 (3.7%) to E5 type, and 2 (1.8%) each to E6, E7, E8, E9, E10, and E11 type. The remaining 10 isolates showed a unique pattern (E12-E21) (Figure 5).

All *K. pneumoniae* isolates from Urology, 75% of isolates from Surgery, and 66.6% from Intensive Care belonged to the E1 type. The E1 type was significantly more frequent in isolates from Surgery (*p* = 0.050) and Intensive Care (*p* = 0.002) compared to Nephrology and in Intensive Care than in Neurology and Internal ward (*p* = 0.004 and 0.002, respectively). E1 type was also more frequently detected in the anal isolates than in urine, blood, and wound isolates (*p* = 0.009, 0.047, and 0.004, respectively) and in respiratory tract isolates compared to wound isolates (*p* = 0.031). E2 was more common among urine isolates compared to anal isolates (*p* = 0.002). In contrast, E3 type was found more frequently observed among isolates from the anus compared to blood (*p* = 0.033).

**Figure 6:**
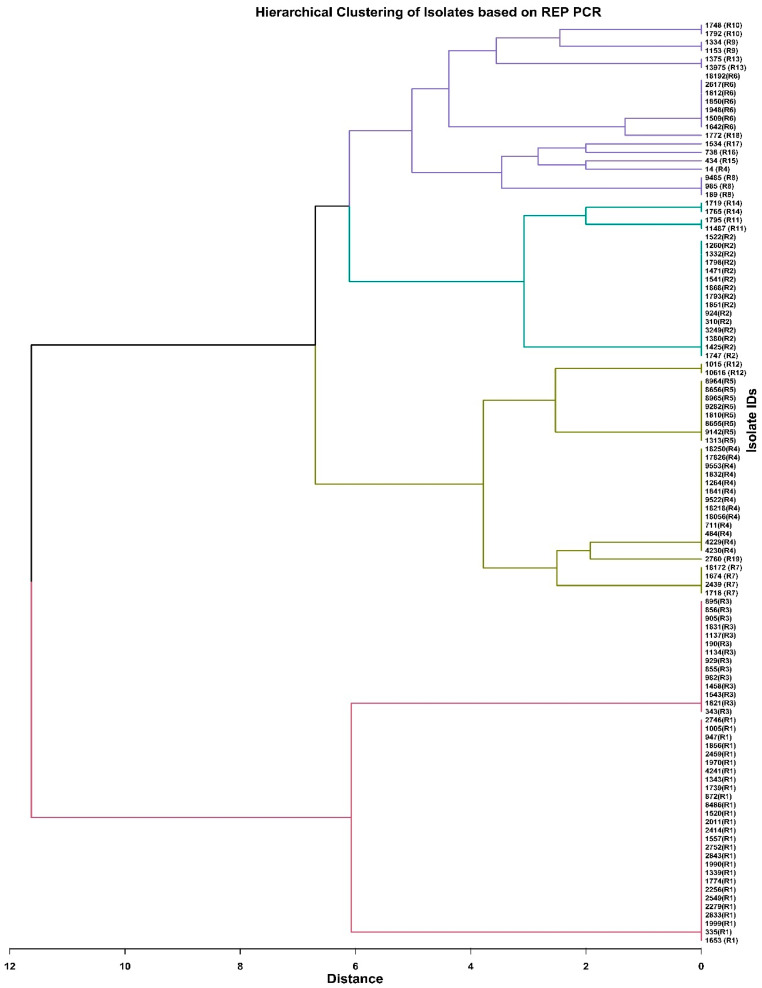
Dendrogram showing the relationship of *K. pneumoniae* isolates based on REP-PCR.

Repetitive element sequence-based PCR (REP-PCR) revealed 19 distinct patterns among *K. pneumoniae* isolates, designated as R1 to R19. The banding patterns ranging from 0.15 to 3.5 kb were obtained using REP-PCR. The most common R types were R1 (27 isolates, 24.8%), R2 (15 isolates, 13.8%), R3 (14 isolates, 12.8%), and R4 (14 isolates, 12.8%). The R types 15–19 were unique, each represented by only one isolate (Figure 6).

R1 and R6 types were significantly more frequent in Surgery than in Internal Medicine ward isolates (*p* = 0.036 and 0.025, respectively). The R1 type was more common in anal isolates than in urine (*p* = 0.000), respiratory tract (*p* = 0.001), blood (*p* = 0.001), and wound isolates (*p* = 0.010). R2 was more frequent among *K. pneumoniae* from the respiratory tract (*p* = 0.028) and wound (*p* = 0.037) than from the anus. The R6 type was more frequent in respiratory tract isolates compared to blood (*p* = 0.034).

The *bla*_SHV-1_, *bla*_CTX-M_ group 1, *mdtK*, *tolC*, *AcrAB* (16.5%); *bla*_SHV-1_, *bla*_CTX-M_ group 1, *tolC*, *AcrAB* (15.6%), and *bla*_SHV-1_, *bla*_CTX-M_ group 1, *bla*_NDM-1_, *mdtK*, *tolC*, *acrAB* (11.9%) were the most common resistance patterns. The isolates with these resistance patterns belonged to different ERIC and REP types (Table 4). The most common virulence gene pattern was *wabG, uge, ureA,* detected in 59.6% of isolates (Table 5).

## 4. Discussion

One of the important mechanisms of antibiotic resistance in *K. pneumoniae* is the expression of extended-spectrum β-lactamases (ESBLs), which confer resistance to cephalosporins and monobactams [16]. Another mechanism of antibiotic resistance in *K. pneumoniae* is the production of carbapenemases that render bacteria resistant to almost all available β-lactams, including the carbapenems. In this study, we investigated the occurrence of genes encoding β-lactamases and efflux pumps among 109 clinical *K. pneumoniae* isolates, consistent with our previous sampling [12]. The β-lactamases are diverse and can be grouped into several families [17]. The TEM-1 enzyme, encoded by a plasmid, was originally found in a single strain of *E. coli* isolated from a blood culture from a patient named Temoniera in Greece, hence, named TEM. The SHV-1 or TEM-1 β-lactamases are highly homologous and differ by only a few amino acid substitutions. TEM-1 β-lactamase is active against aminopenicillins and first-generation cephalosporins such as cephalothin and cephaloridine [18]. Within a few years, the TEM-1 β-lactamase spread worldwide and is now found in various bacterial species. In our study, the *bla*_TEM-1_ gene was detected in 28.4% of isolates, contrasting with the results obtained by Ojdana et al. (Poland) [19], where all ESBL-positive *K. pneumoniae* harbored the *bla*_TEM-1_ gene.

Another common β-lactamase is SHV-1, which is chromosomally encoded in most *K. pneumoniae* isolates, and the *bla*_SHV-1_ gene is responsible for *K. pneumoniae* resistance to ampicillin, amoxicillin, and ticarcillin [20]. Transposable elements such as IS26 mobilize the *bla*_SHV-1_ gene from the *K. pneumoniae* chromosome via transposition [21] into multiple plasmids, facilitating interspecies dissemination [20].

In our study, *bla*_SHV-1_ was the most prevalent β-lactamase gene, consistent with the intrinsic ampicillin resistance of *K. pneumoniae* [20]. Similar results were obtained by Ghenea et al. [22], who showed the presence of the *bla*_SHV-1_ gene in all *K. pneumoniae* isolates from clinical samples in Romania. The high frequency of this gene (85.5%) was also shown by Dehshiri et al. [23] in Iran in *K. pneumoniae* isolates from urinary tract infection, and all hospital isolates in Iraq were positive for this gene [24].

The extensive use of cefotaxime and ceftazidime (third-generation cephalosporins) to treat infections caused by Gram-negative bacilli, resistant to established β-lactams, promoted the development of resistant strains able to overproduce ESBLs, mainly those of class A but also D. Among them, the CTX-M β-lactamases of class A were reported for the first time in the second half of the 1980s. Their dissemination rate among bacteria in most parts of the world has increased dramatically since 1995 [25]. Enzymes of CTX-M type ESBLs are plasmid-encoded and cause transferable resistance to third-generation cephalosporins in Gram-negative bacteria. Infections caused by CTX-M-producing bacteria pose a serious healthcare challenge because limited treatment options for infections caused by these bacteria have led to increased use of carbapenems, which resulted in the emergence and spread of carbapenemase-producing *Enterobacteriaceae* [26]. The CTX-M-type ESBLs are much more genetically diverse. According to Bonnet [25], five major groups of acquired CTX-M enzymes have been identified (the enzymes within each group share more than >94% amino acid sequence similarity, while enzymes belonging to different groups show ≤90% similarity or less). Our study used a multiplex PCR assay [13] to amplify all *bla*_CTX-M_ genes and differentiate the five groups. Our results showed the presence of the *bla*_CTX-M_ genes in 87.2% of *K. pneumoniae* isolates. This indicates that in Poland, these bacteria commonly appear resistant to third-generation cephalosporins. Most isolates showed the *bla_CTX-M_* group 1 gene (83.5%), while in a few (3.7%), the *bla_CTX-M_* group 9 gene was identified. We did not detect *bla*_CTX-M_ genes belonging to the 2, 8, and 25/26 groups. The CTX-M-1 group includes six plasmid-mediated enzymes (CTX-M-1, CTX-M-3, CTX-M-10, CTX-M-12, CTX-M-15, and FEC-1), and the CTX-M-9 group comprises nine enzymes (CTX-M-9, CTX-M-13, CTX-M-14, CTX-M-16, CTX-M-17, CTX-M-19, CTX-M-21, CTX-M-27, and Toho-2) [22]. The study conducted between 1998 and 2000 in 15 hospitals in 10 different cities of Poland revealed the countrywide dissemination of the CTX-M-3 enzyme belonging to CTX-M group 1 [27]. CTX-M-15, a variant of CTX-M-3 previously described in India, has also been observed in Poland [28]. A recent study in Poland also found that *K. pneumoniae* isolates from tracheostomy tubes, which were positive for ESBL production, carried the *bla*_SHV_ and *bla*_TEM_ genes, as well as *bla*_CTX-M_ group 1 genes [29].

The VEB (Vietnamese extended-spectrum β-lactamase), conferring resistance to extended-spectrum cephalosporins and aztreonam, is a plasmid-encoded (pNLT1) ESBL [30]. The presence of the *bla*_VEB-1_ in *K. pneumoniae* strains and other enterobacterial species, *Pseudomonas aeruginosa* [31], and *Acinetobacter baumannii* [32] shows the spread of this resistance gene to other bacteria. In our study, the *bla*_VEB-1_ gene was detected in 11% of isolates, which is consistent with the results obtained for nosocomial isolates by Latifpour et al. [33] in Iran (10.6%) and by Kiratisin et al. [34] in Thailand (10.2%). In our study, most of the *K. pneumoniae* isolates with the *bla*_VEB-1_ gene also harbored the *bla*_SHV-1_, *bla*_CTX-M_ group 1, and *bla*_TEM-1_ genes. On the other hand, the *bla*_VEB-1_ gene was not found in isolates with the *bla*_NDM-1_ gene.

Carbapenems are the last-resort antibiotics used to treat MDR bacterial infections. NDM is a carbapenemase that can hydrolyze most β-lactam drugs, including carbapenems (excluding monobactams), and its activity is not inhibited by new β-lactamase inhibitors such as avibactam and farborbactam [35]. The spread of NDM is challenging to manage, particularly in Pakistan, with a recent report indicating its incidence among carbapenem-resistant isolates to be 53.25% [36]. The genes encoding NDM are usually transmitted through the horizontal transfer of plasmids. The frequency of human clinical isolates of NDM-producing *K. pneumoniae* in Asia, Europe, America, Africa, and Oceania was 64.6%, 20.1%, 9.0%, 5.6%, and 0.4%, respectively [37]. In our study, the *bla*_NDM-1_ gene was detected in 16.5% of isolates. So far, 24 NDM variants have been characterized [38]. In Europe, the NDM-1 type is the most common (99%), and according to Safavi et al. [37], the NDM-1 type was identified in all NDM-producing *K. pneumoniae* isolated from clinical samples in Poland.

KPC is another carbapenemase produced by *K. pneumoniae*, classified as a Class A enzyme. It is widespread in Greece, Italy, and the United States [39]. This study detected the *bla_KPC_* gene only in two isolates (1.8%) obtained from Internal ward and Intensive Care patients. These isolates also harbored *bla*_NDM-1_, *bla*_SHV-1_, and bla_CTX-M_ group 1 genes.

The study by Kamalakar et al. [40] in India did not detect the presence of the *bla*_KPC_ gene in *K. pneumoniae* isolates from clinical samples, while Taha et al. [41] showed the presence of this gene in 4% of *K. pneumoniae* clinical isolates in Egypt.

Our study did not detect imipenem carbapenemases (blaIMP), which are classified as class B enzymes according to Ambler et al. [11] and are frequently reported in India, China, Japan, Russia, and Australia. We also did not find oxacillinases (blaOXA-48), class D enzymes endemic in Turkey and reported in France, Belgium, and North Africa [39]. Different results were reported by Taha et al. [41], who showed the presence of *bla*_OXA-48_ and *bla*_IMP_ genes in 15.5% and 7.5% of *K. pneumoniae* clinical isolates, respectively.

Other factors responsible for the antibiotic resistance of *K. pneumoniae* are efflux pumps. These transport proteins confer resistance by extruding antimicrobials from the bacterial cells, thereby reducing their intracellular concentration and enhancing bacterial survival [42]. Efflux pumps are involved in the β-lactam resistance of *K. pneumoniae*, especially in clinical isolates [43]. In our study, the AcrAB-TolC efflux pump was more common than MdtK, consistent with other reports [44]. AcrAB-TolC is a three-component efflux pump consisting of an outer membrane channel TolC, the inner membrane transporter AcrB, and the periplasmic membrane fusion protein AcrA [45]. Our study showed that all isolates were positive for the *AcrAB* gene, while in seven isolates, the presence of the gene encoding the TolC outer membrane protein was not detected. Similar results were obtained by Wasfi et al. [3], who showed that some *K. pneumoniae* isolates had incomplete AcrAB-TolC pumps. In our study, the *mdtk* gene encoding the multidrug efflux pump system was also present in many isolates (60.5%), while among *K. pneumoniae* isolates from Egyptian hospitals, the percentage of isolates with this gene was lower [3]. In this study, the *mdtk* gene was more frequent in the urine isolates compared to blood and wound, and in respiratory tract isolates compared to wound. The MdtK system transports mainly cationic compounds, including clinically important antibiotics, and thereby, this transporter system confers multidrug resistance to pathogenic bacteria [46].

Endotoxin-related genes (*wabG* and *uge*) were commonly distributed in these isolates. The production of LPS, which protects bacteria from complement-mediated lysis, is regulated by the uridine diphosphate galacturonate 4-epimerase (*uge*) gene. *K. pneumoniae* lacking this gene are less capable of causing sepsis, pneumonia, and urinary tract infections. In various studies, the occurrence of the *uge* gene in *K. pneumoniae* varied widely (41.6% to 86%) [47]. In our study, more than 64% of isolates harbored the *uge* gene. Its prevalence in various clinical specimens was different: the highest in the anus (76.5%), and the lowest in the respiratory tract (43.8%).

The *wabG* gene is important in the biosynthesis of the core lipopolysaccharide (LPS) of *K*. *pneumoniae*. Izquierdo et al. [48] showed that *K. pneumoniae wabG* mutants were avirulent when tested in different animal models (drastic reduction in the colonization ability of *K. pneumoniae* in experimental urinary tract infections and no virulence in an experimental pneumonia model). Furthermore, these mutants were more sensitive to some hydrophobic compounds than the wild-type strains. This study showed that the gene was present in most isolates (88%).

Urease is an important virulence factor for many bacteria [49]. Ammonium from ureolysis provides bacteria with a nitrogen source and acts as an acid neutralizer to protect them from acid stress damage. Urease activity is required for *K. pneumoniae* to colonize the mouse gastrointestinal tract [50], while in the urinary tract with abundant urea, urease activity may promote the formation of urinary stones. Due to its enzymatic activity, urease has a toxic effect on human cells [51]. The urease activity of *Klebsiella* spp. correlated with developing hepatic encephalopathy [52].

The ureDABCEFG operon encodes urease; the *ureABC* genes are responsible for the structural subunits, and the *ureDEFG* genes encode the accessory proteins binding nickel ions, being enzyme activators [53]. Our study showed that the *ureA* gene encoding the γ subunit [54] of the structural unit of urease was present in 94.5% of isolates.

In this study, we also investigated the frequency of a gene encoding the most important virulence factor in *K. pneumoniae* siderophores that was not identified in the previous study. Siderophores enable the bacteria to obtain iron by transporting ferric ions into the cell, thereby promoting the growth and metabolism of bacteria [55].

Aerobactin is closely associated with invasive infections caused by *K. pneumoniae* and is the main siderophore responsible for hypervirulence [56]. There is evidence that aerobactin primarily transports iron from the host cells and may increase the iron content in *K. pneumoniae* even under iron deficiency conditions, which was considered the most important virulence factor of *K. pneumoniae* [57]. The *iucB* was identified as the most important gene encoding aerobactin, closely associated with the hypervirulence of *K. pneumoniae* [55].

**Figure 7:**
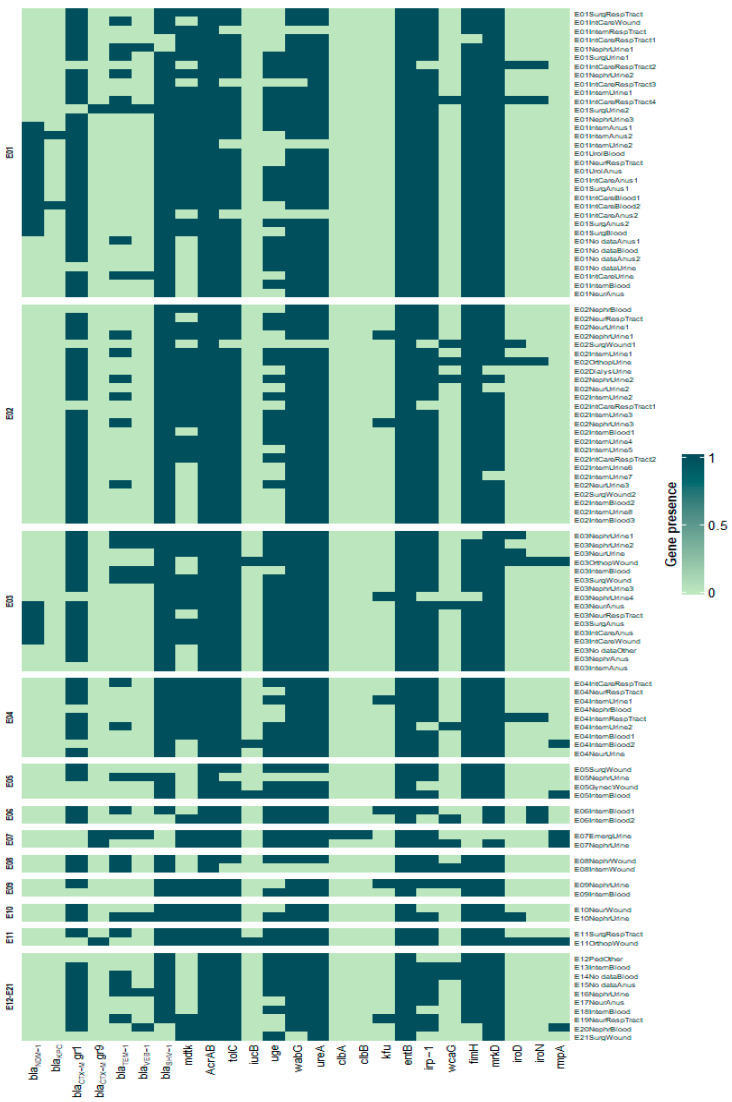
Heatmap representation of the occurrence of genes coding resistance and virulence mechanisms in clinical isolates of *Klebsiella pneumoniae* belonging to different ERIC types.

In our study, the *iucB* gene was detected in only 4 (3.7%) isolates from blood (Internal ward) and wound (Orthopedics), and was more frequently observed in wound isolates than in blood. Statistical analysis showed that the occurrence of the hypervirulence genes that were identified in our previous study—*rmpA* (regulator of mucoid phenotype A), *iroD* (encoding salmochelin biosynthesis), *iroN* (encoding salmochelin receptor gene) [12], and *iucB* identified in this study—was significantly higher in Orthopedics compared to other hospital units. Two *K. pneumoniae* isolates with *iucB* from wounds in Orthopedics also harbored *rmpA*, *iroD,* and *iroN* genes. In contrast, the genes encoding salmochelin (*iroD* and *iroN*) were not detected in isolates with *iucB* from blood in the Internal ward (Figure 7).

Isolates from the Orthopedics showed a wide range of virulence possibilities due to the production of several siderophores that facilitate the colonization of tissues in case one of them is neutralized by the host [58]. In the investigated population of *K. pneumoniae*, isolates with *iucB* did not show the *kfu* gene encoding the iron acquisition system and the *wcaG* gene that encodes capsular fucose synthesis and enhances the ability of bacteria to evade phagocytosis. All isolates with *iucB* had the *bla*_SHV-1_ gene (Figure 7).

To study the epidemiological relatedness among *K. pneumoniae* isolates, we used and compared two methods of typing based on PCR. ERIC-PCR was a better tool for discriminating *K. pneumoniae* isolates than REP-PCR. Twenty-one ERIC profiles (E-types) were observed, while repetitive element sequence-based PCR (rep-PCR) revealed only nineteen patterns of *K. pneumoniae* isolates. More than 30% of isolates belonged to type E1 and about 23% to type E2. The other types (E3 and E4) comprised about 15% and above 8%, respectively. The remaining types included fewer isolates. In our study, *K. pneumoniae* isolates were less diverse compared to those evaluated by Kundu et al. [59], who studied *K. pneumoniae* clinical isolates in India and obtained 40 ERIC types, or Mehr et al. [60], who obtained 32 different ERIC types among clinical isolates in Iran. The ERIC genotypes in our study showed different resistance and virulence genes. The analysis of the distribution of resistance and virulence genes within each ERIC type showed that isolates belonging to the same ERIC type differed in their virulence gene profiles and antibiotic resistance patterns and were collected from different hospital departments and clinical sources. This suggests that there was no clear evidence of a single clonal outbreak during the study period, but rather the presence of genetically diverse isolates circulating in the hospital. The *bla*_NDM-1_ gene was identified in the E1 and E3 types, while the *bla*_VEB-1_ gene was not detected in the E2 type. The *kfu* gene was not present in isolates belonging to the E1 type. The isolates with genes that are diagnostic biomarkers for hypervirulent *K. pneumoniae* (*rmpA*, *iroD*, *iroN*, *iucB*) belonged to different ERIC types (E3, E4, E5, and E11).

## 5. Conclusions

*K. pneumoniae* is a very important nosocomial pathogen characterized by great diversity in traits determining virulence and the ability to acquire genes encoding antibiotic resistance mechanisms. This makes infections caused by *K. pneumoniae* difficult to treat and challenges healthcare.

Isolates of *K. pneumoniae* analyzed in the present study, obtained from various hospital wards and materials, showed numerous and diverse antibiotic resistance and virulence genes and their combinations. The frequency of individual genes showed differences between the wards, and to a lesser extent, between clinical materials. Efflux pump genes *Acr*AB and *tol*C and β-lactamase genes *bla*_CTX-M_ group 1 and *bla*_SHV-1_ were the most common among the resistance genes, while *wab*G and *ure*A were the most frequent among the virulence genes (in addition to the earlier detected *entB*, *mrkD,* and *fimH* genes). The presence of the hypervirulence gene *iucB* strongly correlated with the presence of another hypervirulence gene, *rmpA,* detected earlier in the same isolates. ERIC-PCR genotyping revealed 21 genotypes. The antibiotic resistance and virulence patterns were not related to the genotype. The common presence of β-lactamases, including ESBL, in *K. pneumoniae* isolates indicates that β-lactam antibiotics should not be used for the treatment of infections caused by this microorganism without prior assessment of their sensitivity to this group of antibiotics. The frequent presence of multiple virulence genes, including those conferring hypervirulence, shows that these isolates can cause severe infections. Future research should include a larger number of hospitals and, consequently, a greater number of *K. pneumoniae* isolates in order to obtain broader knowledge on the resistance and virulence of *K. pneumoniae* isolates in Poland. Furthermore, to gain deeper insight into the genetic diversity of *K. pneumoniae* isolates, future studies should also use advanced genomic sequencing techniques.

## Figures and Tables

**Table 1:** The presence of genes coding β-lactam resistance in *K. pneumoniae* isolates from various clinical materials and hospital wards. The results in brackets show the number of isolates carrying the gene and the percentage of the gene carriers among the isolates from a certain material or hospital ward.

Resistance Genes	Isolates with Gene (*n*, %)		
	Total	Source of Isolates	Hospital Wards
*bla*_SHV-1_	101 (92.7)	Respiratory tract (15, 92.3), anus (17, 100), blood (20, 90.9), urine (36, 92.3), wound (11, 84.6), no data (2, 100)	Internal (30, 96.7), Nephrology (17, 85), Neurology (14, 100), Surgery (11, 84.6), Intensive Care (14, 93.3), Orthopedics (3, 100), Urology (2, 100), Pediatrics (1, 100), Dialysis Station (1, 100), Gynecology and Obstetrics (1, 100), No data (6, 100)
*bla*_CTX-M_ group 1	91 (83.5)	Respiratory tract (14, 87.5), anus (16, 94.1), blood (17, 77.3), urine (34, 87.2), wound (9, 69.2), no data (1, 50)	Internal (27, 87.1), Nephrology (16, 80), Neurology (14, 100), Surgery (10, 76.9), Intensive Care (13, 86.7), Orthopedics (2, 66.7), Urology (2, 100), Dialysis Station (1, 100), No data (6, 100)
*bla*_CTX-M_ group 9	4 (3.7)	Urine (3, 7.7), wound (1, 7.7)	Nephrology (1, 5), Surgery (1, 7.7), Emergency Department (1, 100), Orthopedics (1, 33.3),
*bla*_TEM-1_	31 (28.4)	Respiratory tract (4, 25), anus (2, 11.8), blood (3, 13.7), urine (18, 46.1), wound (4, 30.8)	Nephrology (11, 55), Internal (6, 19.6), Surgery (4, 30.8), Neurology (2, 14.3), Emergency Department (1, 100), Intensive Care (4, 26.7), No data (3, 50)
*bla*_NDM-1_	18 (16.5)	Anus (10, 58.8), blood (4, 18.2), respiratory tract (2, 12.5), urine (1, 2.6), wound (1, 7.7)	Internal (3, 9.7), Neurology (3, 21.4), Surgery (4, 30.8), Intensive Care (6, 40), Urology (2, 100)
*bla*_KPC_	2 (1.8)	Anus (1, 5.9), blood (1, 4.6)	Internal (1, 3.2), Intensive Care (1, 6.7)
*bla*_VEB-1_	12 (11.0)	Urine (9, 23.1), blood (2, 9.1), wound (1, 7.7)	Nephrology (7, 35), Internal (1, 3.2), Surgery (2, 15.4), Emergency Department (1, 100), Intensive Care (1, 6.7)

**Table 2:** The presence of genes coding efflux pumps in *K. pneumoniae* isolates from various clinical materials and hospital wards. The results in brackets show the number of isolates carrying the gene and the percentage of the gene carriers among the isolates from a certain material or hospital ward.

Genes	Isolates with Gene (*n*, %)		
	Total	Source of Isolates (*n*, %)	Hospital Wards (*n*, %)
*AcrAB*	109 (100)	Blood (22, 100), urine (39, 100), anus (17, 100), respiratory tract (16, 100), wound (13, 100), no data (2, 100)	Internal (31, 100), Nephrology (20, 100), Surgery (13, 100), Neurology (14, 100), Intensive Care (15, 100), Gynecology and Obstetrics (1, 100), Orthopedics (3, 100), Pediatrics (1, 100), Dialysis Station (1, 100), Emergency Department (1, 100), Urology (2, 100), No data (7, 100)
*tolC*	102 (93.6)	Blood (22, 100), urine (37, 94.9), anus (16, 94.1), respiratory tract (14, 87.5), wound (11, 84.6), no data (1, 50)	Internal (28, 90.3), Nephrology (19, 94.0), Surgery (12, 93.3), Neurology (14, 100), Intensive Care (13, 86.7), Orthopedics (3, 100), Gynecology and Obstetrics (1, 100), Pediatrics (1, 100) Dialysis Station (1, 100), Emergency Department (1, 100), Urology (2, 100), No data (6, 100)
*mdtk*	66 (60.5)	Blood (10, 45.5), urine (30, 76.9), anus (9, 52.9), respiratory tract (12, 75.0), wound (5, 38.5)	Internal (16, 51.6), Nephrology (16, 80.0), Surgery (10, 76.9), Neurology (8, 57.1), Intensive Care (10, 66.7), Orthopedics (2, 66.7), Dialysis Station (1, 100), Emergency Department (1, 100), Urology (2, 100)

**Table 3:** The presence of virulence genes in *K. pneumoniae* isolates from various clinical materials and hospital wards. The results in brackets are presented as the number of isolates carrying the gene and the percentage of the gene carriers among the isolates from a certain material or hospital ward.

Virulence Genes	Isolates with Gene (*n*, %)		
	Total	Source of Isolates (*n*)	Hospital Wards (*n*)
Endotoxin-related genes			
*wabG*	101 (92.7)	Blood (22, 100), urine (37, 94.9), anus (16, 94.1), respiratory tract (14, 87.5), wound (10, 76.9), no data (2, 100)	Internal (28, 90.3), Nephrology (19, 95.0), Surgery (11, 84.6), Neurology (14, 100), Intensive Care (13, 86.7), Gynecology and Obstetrics (1, 100) Orthopedics (3, 100), Pediatrics (1, 100), Dialysis Station (1, 100), Emergency Department (1, 100), Urology (2, 100), No data (7, 100)
*uge*	70 (64.2)	Blood (13, 59.1), urine (27, 69.2), anus (13, 76.5), respiratory tract (7, 43.8), wound (8, 61.5), no data (2, 100)	Internal (19, 61.3), Nephrology (13, 65.0), Surgery (9, 69.2), Neurology (7, 50.0), Intensive Care (8, 53.3), Orthopedics (3, 100), Pediatrics (1, 100), Dialysis Station (1, 100), Emergency Department (1, 100), Urology (1, 50), No data (7, 100)
Siderophore gene			
*iucB*	4 (3.7)	Blood (2, 9.1), wound (2, 15.4)	Internal (2, 6.45), Orthopedics (2, 66.7)
Subunit of urease gene			
*ureA*	103 (94.5)	Blood (22, 100), urine (37, 94.9), anus (16, 94.1), respiratory tract (15, 93.8), wound (11, 84.6), no data (2, 100)	Internal (28, 90.3), Nephrology (19, 95.0), Surgery (12, 92.3), Neurology (14, 100), Intensive Care (14, 93.3), Orthopedics (3, 100), Gynecology and Obstetrics (1, 100), Pediatrics (1, 100), Dialysis Station (1, 100), Emergency Department (1, 100), Urology (2, 100), No data (7, 100)

**Table 4:** The prevalence of genes coding resistance to β-lactams and efflux pumps in *K. pneumoniae* isolates belonging to different ERIC and REP types. The genotypes without numbers in brackets include only one isolate.

No. of Isolates	Genes Coding Resistance to β-Lactams (*bla* Genes)									Genes Coding Efflux Pumps			Profile of	
	_SHV-1_	_CTX-M gr1_	_CTX-M gr9_	_TEM-1_	_VEB-1_	_NDM-1_	_KPC_	_IMP_	_OXA-48_	*mdt*	*tolC*	*AcrA*	ERIC (*n*)	REP (*n*)
18	+	+	−	−	−	−	−	−	−	+	+	+	E1(3), E2(8), E3, E4(4), E9, E10	R1(2), R2(4), R3(7), R6, R7, R9, R10, R14
17	+	+	−	−	−	−	−	−	−	−	+	+	E1(4), E2(8), E3(3), E4	R1(2), R2, R3, R4(4), R5(6), R12, R14, R19
13	+	+	−	−	−	+	−	−	−	+	+	+	E1(9), E3(4)	R1(11), R6, R7
10	+	+	−	+	−	−	−	−	−	+	+	+	E1(3), E2(4), E4, E11, E19	R1, R2, R3(2), R4(2), R6(3), R18
7	+	+	−	+	−	−	−	−	−	−	+	+	E1(2), E2, E6, E8, E14, E15	R2, R4, R7(2), R8, R10, R13
7	+	−	−	−	−	−	−	−	−	−	+	+	E1(2), E3, E4, E5, E6, E12	R1(2), R2, R4(2), R6(2)
5	+	−	−	−	−	−	−	−	−	+	+	+	E2(2), E3, E4, E9	R1, R2(2) R3, R5
3	+	+	−	+	+	−	−	−	−	−	−	−	E3(2), E10	R2, R4, R5
2	+	+	−	−	−	+	+	−	−	+	+	+	E1(2)	R1(2)
2	+	+	−	+	+	−	−	−	−	−	+	+	E3, E16	R3, R12
2	−	+	−	+	+	−	−	−	−	+	+	+	E1, E6	R11, R13
2	+	+	−	+	−	−	−	−	−	+	+	+	E2, E4	R3, R4
2	−	+	−	−	−	−	−	−	−	+	+	+	E1, E6	R2, R13
2	+	+	−	−	−	−	−	−	−	−	+	+	E13, E18	R4, R11
1	+	+	−	+	+	−	−	−	−	+	+	+	E1	R4
1	−	−	+	+	+	−	−	−	−	+	+	+	E7	R16
1	+	+	−	+	+	−	−	−	−	+	+	+	E3	R2
1	−	+	−	−	+	−	−	−	−	+	+	+	E20	R8
1	+	+	−	+	+	−	−	−	−	−	+	+	E1	R4
1	+	+	−	−	−	+	−	−	−	−	+	+	E3	R1
1	−	+	−	−	−	−	−	−	−	+	+	+	E7	R8
1	+	+	−	+	+	−	−	−	−	−	−	+	E5	R9
1	+	+	−	−	−	−	−	−	−	+	+	+	E3	R2
1	+	+	−	−	−	+	−	−	−	+	−	+	E1	R1
1	−	−	−	−	−	−	−	−	−	+	+	+	E21	R15
1	−	+	−	−	−	−	−	−	−	−	+	+	E5	R1
1	+	+	−	−	−	+	−	−	−	−	−	+	E1	R1
1	+	+	−	+	−	−	−	−	−	−	−	+	E8	R2
1	+	+	−	−	−	−	−	−	−	−	−	+	E1	R3
1	+	+	−	−	−	−	−	−	−	+	−	+	E1	R1
1	+	−	−	−	−	−	−	−	−	−	−	+	E2	R1

**Table 5:** The prevalence of virulence genes in *K. pneumoniae* isolates belonging to different ERIC and REP genotypes. The number of isolates that are characterized by a specified pattern of the presence of genes coding for virulence factors and belonging to certain genotypes is presented in brackets. The genotypes without numbers in brackets include only one isolate.

No. of Isolates	Virulence Genes				Profile of:	
	*wabG*	*uge*	*iucB*	*ureA*	ERIC	REP
4	+	+	+	+	E3, E4, E5, E11	R1, R4(2), R17
65	+	+	−	+	E1(18), E2(12), E3(14), E4(5), E5(2), E6(2), E7(2), E8(1), E9(1), E10(1), E11(1), E12(1), E13(1), E14(1), E15(1), E16(1), E18(1)	R1 (16), R2(11), R3(9), R4(10), R5(1), R6(5), R7(3), R8(2), R11(1), R12(1), R13(2), R14(2), R16(1), R19(1)
32	+	−	−	+	E1(11), E2(12), E3(1), E4(3), E9(1), E10(1), E17(1), E19(1), E20(1)	R1(6), R2(3), R3(4), R4(2), R5(7), R6(2), R7(1), R8(1), R9(1), R10(2), R11(1), R12(1), R18(1)
1	−	−	−	+	E1	R3
1	−	+	−	−	E21	R15
6	−	−	−	−	E1(3), E2, E5, E8	R1(4), R2, R9

## Data Availability

The original contributions presented in this study are included in the article. Further inquiries can be directed to the corresponding author.

## Supplementary Materials

The following supporting information can be downloaded at: https://www.mdpi.com/article/10.3390/pathogens14070648/s1, Table S1: Primers used for PCR; Figure S1: Number of resistance genes: coding β-lactamases and efflux pumps in *Klebsiella pneumoniae* isolates obtained from various hospital wards; Figure S2: Number of resistance genes: coding β-lactamases and efflux pumps in *Klebsiella pneumoniae* isolates obtained from various clinical materials; Figure S3: Number of virulence genes: coding non-hypervirulence and hypervirulence mechanisms in *Klebsiella pneumoniae* isolates obtained from various hospital wards; Figure S4: Number of virulence genes: coding non-hypervirulence and hypervirulence mechanisms in *Klebsiella pneumoniae* isolates obtained from various clinical materials.
