# Supplementary material for: Antibiotic Resistance, Virulence Genes, and Molecular Diversity of Clinical Klebsiella pneumoniae Isolates from Patients of District Hospital in Central Poland
Source: Pathogens. 2025 Jun 30;14(7):648. doi: 10.3390/pathogens14070648 (PMC12298917; doi:10.3390/pathogens14070648)
Supplement: Supplementary file 1 [file pathogens-14-00648-s001.zip › pathogens-3684952-supplementary.pdf]

Table S1. Primers used for PCR.

Target gene	Primer sequences (5' → 3')	Length (bp)	Reference
bla$_{SHV-1}$	F: CCTCATTCAGTTCCGTTTCC	389	Latifpour et al. [33]
	R: CCGCGTAGGCATGATAGAAA		
bla$_{VEB-1}$	F: GGGTATTCCAATCCTTGTGC	250	
	R: CCCTCAAGACCTTTTGCCTA		
bla$_{TEM-1}$	F: GCTATGTGGCGCGGTATTAT	189	
	R: AAGTTGGCCGCAGTGTTATC		
bla$_{CTX-M}$ group1	F: GCGTGATACCACTTCACCTC	260	Xu et al. [12]
	R: TGAAGTAAGTGACCAGAATC		
bla$_{CTX-M}$ group2	F: TGATACCACCACGCCGCTC	341	
	R: TATTGCATCAGAAACCGTGGG		
bla$_{CTX-M}$ groups 8 and 25/26	F: CAATCTGACGTTGGGCAATG	207	
	R: ATAACCGTCGGTGACAATT		
bla$_{CTX-M}$ group9	F: ATCAAGCCTGCCGATCTGGTTA	293	
	R: GTAAGCTGACGCAACGTCTGC		
bla$_{NDM-1}$	F: GGTTTGGCGATCTGGTTTTC	621	Poirel et al.
	R: CGGAATGGCTCATCACGATC		
bla$_{KPC}$	F: CGTCTAGTTCTGCTGTCTTG	798	
	R: CTTGTCATCCTTGTTAGGCG		
bla$_{OXA-48}$	F: GCGTGGTTAAGGATGAACAC	438	
	R: CATCAAGTTCAACCCAACCG		
bla$_{IMP}$	F: GGAATAGAGTGGCTTAAYTCTC	232	
	R: GGTTTAAYAAAACAACCACC		
AcrAB	F: ATCAGCGGCCGGATTGGTAAA	312	Wasfi et al. [3]
	R: CGGGTTCGGGAAAATAGCGCG		
tolC	F:ATCAGCAACCCCGATCTGCGT	527	
	R: CCGGTGACTTGACGCAGTCCT		
mdtk	F: GCGCTTAACTTCAGCTCA	453	
	R: GATGATAAATCCACACCAGAA		
iucB	F: ATGTCTAAGGCAAACATCGT	948	Hsieh et al.
	R: TTACAGACCGACCTCCGTGA		
wabG	F: CGGACTGGCAGATCCATATC	683	Yu et al. 2006 [5]
	R: ACCATCGGCCATTTGATAGA		
uge	F: GATCATCCGGTCTCCCTGTA	535	
	R: TCTTCACGCCTTCCTTCACT		
ureA	F: GCTGACTTAAGAGAACGTTATG	337	Lee et al. [54]
	R: GATCATGGCGCTACCT(C/T)A		
REP-1	F: III ICG ICG ICA TCI GGC		Versalovic et al. [13]
REP-2	R: ICG ICT TATCIG GCC TAC		
ERIC-1	F: CACTTAGGGGTCCTCGAA TGTA		
ERIC-2	R: AAGTAAGTGACTGGGG TGAGCG		

References

1. Poirel, L., Walsh, T. R., Cuvillier, V. & Nordmann, P. Multiplex PCR for detection of acquired carbapenemase genes. *Diagn. Microbiol. Infect. Dis.* **70**, 119–123. http://dx.doi.org/10.1016/j.diagmicrobio.2010.12.002 (2011).
2. Hsieh, P. F., Lin, T. L., Lee, C. Z., Tsai, S. F. & Wang, J. T. Serum-induced iron-acquisition systems and TonB contribute to virulence in *Klebsiella pneumoniae* causing primary pyogenic liver abscess. *J. Infect. Dis.* **197**, 1717–1727. DOI: 10.1086/588383 (2008).

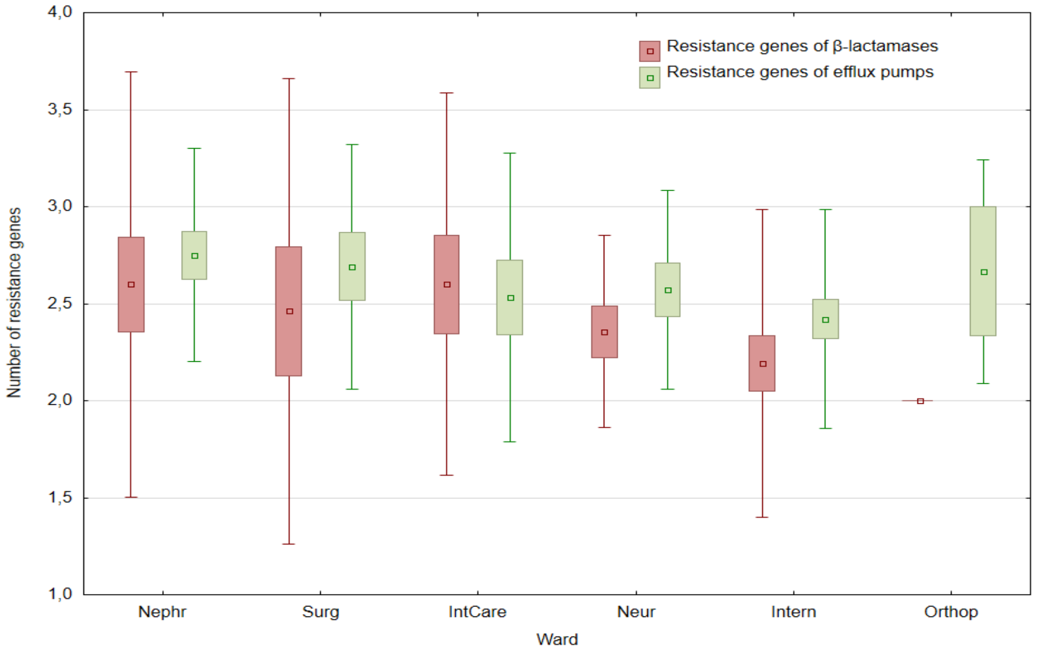

Figure S1. Number of resistance genes: coding β-lactamases and efflux pumps in *Klebsiella pneumoniae* isolates obtained from various hospital wards, arithmetic means (point), S.E. (box), S.D. (whiskers), no significant differences found (post-hoc Tukey's Honest Significant Difference (HSD) test, p≤0.05).

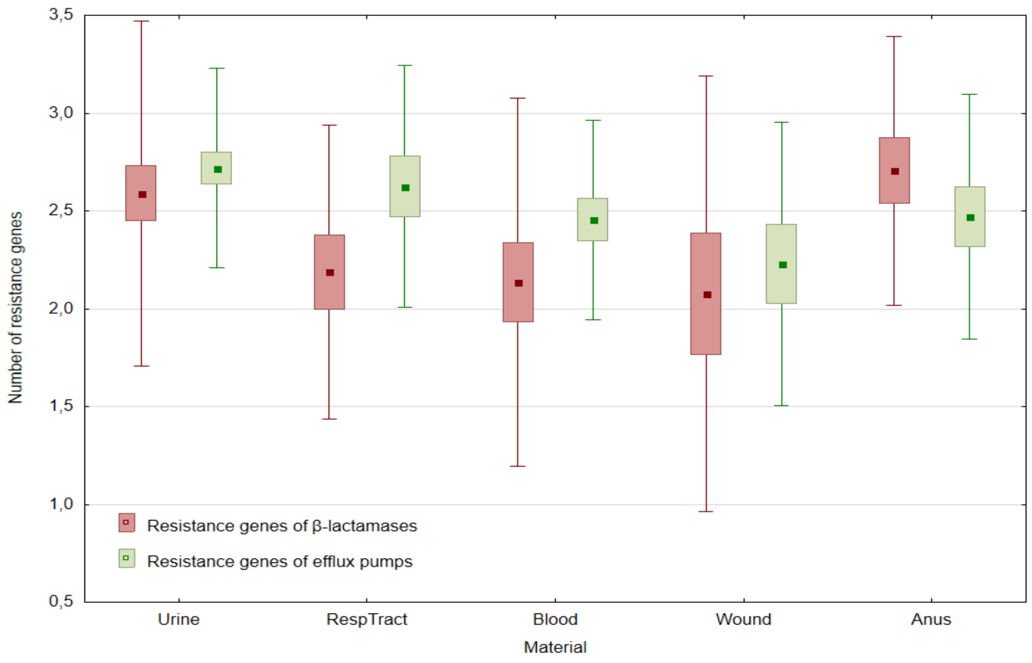

Figure S2. Number of resistance genes: coding β-lactamases and efflux pumps in *Klebsiella pneumoniae* isolates obtained from various clinical materials, arithmetic means (point), S.E. (box), S.D. (whiskers), no significant differences found (post-hoc Tukey's Honest Significant Difference (HSD) test, p≤0.05).

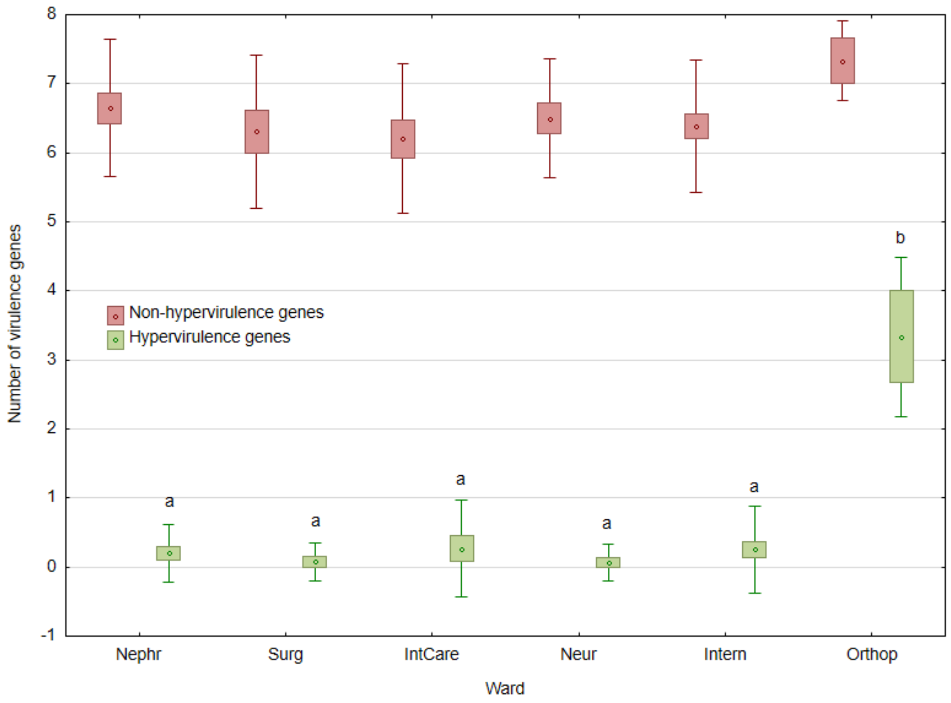

Figure S3. Number of virulence genes: coding non-hypervirulence and hypervirulence mechanisms in *Klebsiella pneumoniae* isolates obtained from various hospital wards, arithmetic means (point), S.E. (box), S.D. (whiskers), significant differences indicated with different letter superscripts (post-hoc Tukey's Honest Significant Difference (HSD) test, p≤0.05).

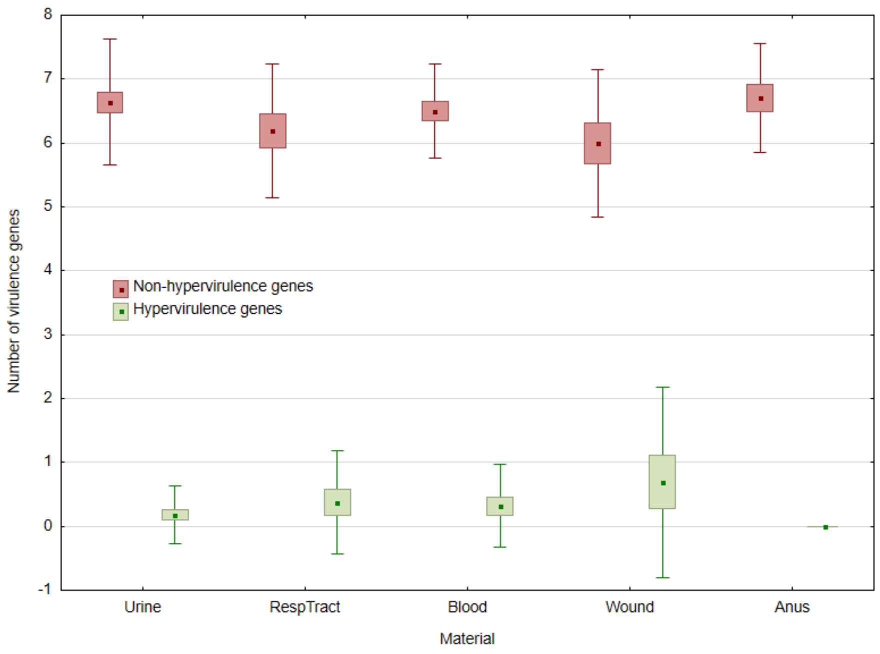

Figure S4. Number of virulence genes: coding non-hypervirulence and hypervirulence mechanisms in *Klebsiella pneumoniae* isolates obtained from various clinical materials, arithmetic means (point), S.E. (box), S.D. (whiskers), no significant differences found (post-hoc Tukey's Honest Significant Difference (HSD) test, $p \leq 0.05$).
